# Microsatellite polymorphism in the *Heme oxygenase-1* gene promoter is associated with dermal collagen density in Japanese obese male subjects

**DOI:** 10.1371/journal.pone.0199994

**Published:** 2018-07-19

**Authors:** Ai Ibuki, Takeo Minematsu, Mikako Yoshida, Shinji Iizaka, Masaru Matsumoto, Junko Sugama, Hiromi Sanada

**Affiliations:** 1 Department of Biological Science and Nursing, School of Medicine, Yokohama City University, Kanagawa, Japan; 2 Department of Gerontological Nursing/ Wound Care Management, Graduate School of Medicine, The University of Tokyo, Tokyo, Japan; 3 Department of Skincare Science, Graduate School of Medicine, The University of Tokyo, Tokyo, Japan; 4 Department of Imaging Nursing Science, Graduate School of Medicine, The University of Tokyo, Tokyo, Japan; 5 School of Nutrition, College of Nursing and Nutrition, Shukutoku University, Chiba, Japan; 6 Advanced Health Care Science Research Unit Innovative Integrated Bio-Research Core Institute for Frontier Science Initiative, Kanazawa University, Ishikawa, Japan; University of Missouri Health Care, UNITED STATES

## Abstract

We previously reported elevated oxidative stress-related mechanical vulnerabilities of the skin as sparse distributions of hyperechoic areas. Although this helped establish a personalized skin care system to prevent skin disorders related to mechanical stress, obesity-related skin vulnerability involves individual differences. Here, we hypothesized that individual differences are caused by polymorphisms of GT repetitive sequences in the *heme oxygenase1* (*HMOX1*) promoter region, which encodes an antioxidant enzyme. This cross-sectional study enrolled healthy male volunteers in a walking classroom aimed at weight control. Subjects with a body mass index <25 kg/m^2^ were classified as non-obese and those with body mass index ≥25 kg/m^2^ were classified as obese. Subject skin was categorized into sparse dermis or normal groups according to the distribution of hyperechoic areas by high-resolution skin ultrasonography (20 MHz). Genomic DNA and mRNA extracted from three body hairs with attached follicle cells were used to analyze GT repetitive sequences of the *HMOX1* promoter, *HMOX1* mRNA expression levels, and oxidative stress levels (8-hydroxy-2’-deoxyguanosine). Classifications of GT repetitive sequence of *HMOX1* promoter were Short (<27 times) and Long (≥27 times). Higher numbers of subjects with sparse dermis were in the obese group compared with the non-obese group. In obese subjects, the number of subjects that had the Long allele of the *HMOX1* promoter with sparse dermis was significantly higher compared with the normal group, whereas no association was observed between the polymorphism and ultrasonographic features in non-obese subjects. Thus, *HMOX1* polymorphisms detected a risk of low collagen density in Japanese obese male subjects.

## Introduction

Obesity is one of the most common health problems worldwide. The Japan Society for the Study of Obesity defined obesity as a body mass index (BMI) ≥25 kg/m^2^ [1]. In Japan, the prevalence of obesity is increasing and is currently 28.6% in men and 20.3% in women aged ≥20 years [2]. Obesity is associated with visceral diseases as well as skin disorders related to the mechanical properties of skin [3, 4]. Decreased mechanical strength is considered a risk for pressure ulcer [5, 6] and skin tears [7] that are caused by external forces. Skin wounds seriously impair the quality of life of obese individuals. Therefore, it is necessary to elucidate the mechanism of decreased mechanical strength to establish prevention and care.

Mechanical strength of the skin is primarily dependent on the dermal matrix, which is mainly composed of type I collagen [8]. A histological study by Akase *et al*. [9] revealed that the density of collagen fibers in the dermis of obese mice were reduced compared with control mice. Other studies reported decreased numbers of fibroblasts and *Col1a1* mRNA expression in high-fat diet-induced obese mice [10, 11]. These findings suggest that obesity inhibits the synthesis and promotes the degradation of dermal matrixes resulting in decreased collagen density in the dermis. We previously reported that the elevated oxidative stress-induced overexpression of matrix metalloproteinases degraded dermal collagen and promoted dermal vulnerability in obese mice [4]. Decreased collagen density and increased oxidative stress were similarly observed in healthy human volunteers [12, 13]. However, approximately 40% of overweight and 10% of obese subjects had normal collagen density suggesting individual differences in reduced collagen density are associated with the progression of obesity [12].

In this study, we focused on *heme oxygenase 1* (*HMOX1*) polymorphisms. HMOX1 is an enzyme involved in the defense system against oxidative stress. HMOX1 catalyzes heme into biliverdin, F^2+^, and CO, which inhibit the Fenton reaction and decrease free radicals [14, 15]. The human *HMOX1* gene has a GT dinucleotide repeat sequence in the promoter region. A GT repeat is the most frequent of the simple repeats scattered throughout the human genome, and many of these exhibit length polymorphisms [16, 17]. A previous study reported that the number of GT repeats was negatively correlated with *HMOX1* transcriptional activity [17]. Thus, *HMOX1* polymorphisms define individual differences in antioxidant capacity. Furthermore, *HMOX1* polymorphisms are associated with emphysema, coronary artery disease, and type 2 diabetes mellitus [18–21]. Based on these findings, we hypothesized that *HMOX1* polymorphisms are associated with individual differences regarding decreased dermal collagen in obese individuals.

The aim of this study was to examine the association of *HMOX1* promoter polymorphisms with the skin expression of *HMOX1* and with dermal collagen density in Japanese obese subjects.

## Materials and methods

### 1. Study design and setting

This cross-sectional observation study was conducted at the Wellness Promotion Science Center of Kanazawa University (Ishikawa, Japan) from May to August 2013.

### 2. Subjects

The subjects (20–64 years old) were participants of a walking class aimed at weight control or health promotion. Subjects included male individuals from whom body hairs were easily collected from the thigh. Female subjects were excluded because it was difficult to collect body hairs. Subjects with systemic or chronic inflammatory skin disorders were excluded. Subjects were classified according to their BMI into a non-obese group (<25 kg/m^2^) or obese group (≥25 kg/m^2^).

### 3. Characteristics of the study subjects

We measured subjects’ height, age, past and present smoking habits, past weight change, and health conditions (diabetes, hyperglycemia, hypertension, hyperlipidemia, and medication). Subject body weight and body fat ratio (%) were measured by a multi-frequency body composition monitor (Tanita, Tokyo, Japan), and BMI (kg/m^2^) and waist circumference was measured using a measuring tape.

### 4. Examination site

We examined the left thigh at a height of the middle point of the greater trochanter and the kneecap of subjects because of the higher accumulation of subcutaneous fat, a lower effect of photo aging, and abundant body hair.

### 5. Dermal collagen density

**Fig 1:**
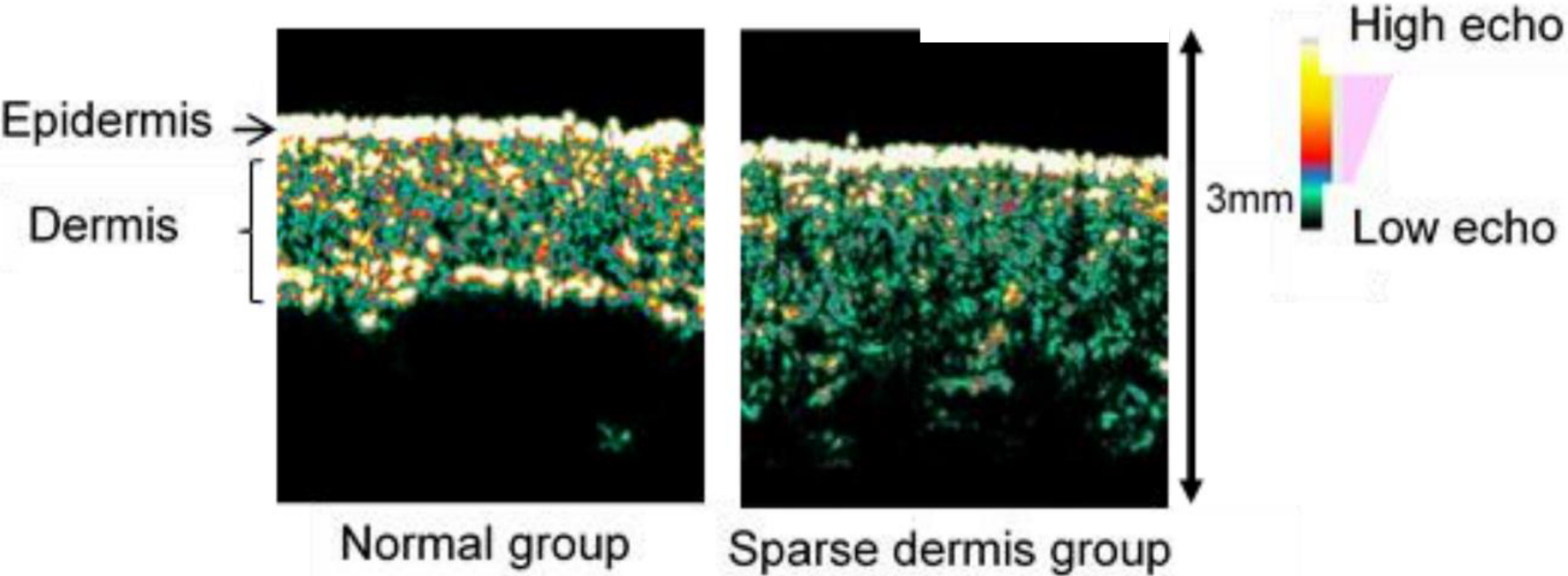
Ultrasound images. We classified ultrasound images into two types. Left panel: normal group where the hyperechoic area is present in the entire dermis; and Right panel: sparse dermis group where the hyperechoic area is absent in part of or the whole dermis.

High-resolution ultrasonography with a 20-MHz linear probe (Dermascan C, Cortex Technology, Hadsund, Denmark) was used to evaluate dermal collagen density. The resolution was 60×130 μm, and ultrasound penetration was a depth of 10 mm by ultrasonography according to the manufacturer’s information. Scanning conditions were fixed at 3 for gain key and 10 for gain profile level. Collagen fibers were detected as hyperechoic signals whose intensity represented collagen density [22, 23]. We classified the ultrasound images into normal and sparse groups according to the distribution of hyperechoic signals as previously described [12] (Fig 1). In this study, inter-rater reliability for this classification was κ = 0.72 between two researchers.

### 6. Body hair sampling

After shaving hair shafts with a surgical clipper (3M, St. Paul, MN, USA), three body hairs to which follicular cells were attached were harvested with a pair of tweezers for biochemical analyses including polymorphisms (GT repeats) of the *HMOX1* promoter, 8-hydroxy-2’-deoxyguanosine (8OHdG) as a parameter for oxidative stress levels, and *HMOX1* mRNA expression levels. The collected samples were stored in 1 mL of RNA later (Thermo Fisher Scientific, Waltham, MA, USA) at 4°C and RNA was extracted within 1 week. Total RNA and genomic DNA were extracted using an All Prep DNA/RNA Micro Kit (Qiagen, Venlo, Netherlands) according to the manufacturer’s instructions. Some subjects in which sufficient genomic DNA and total RNA could not be extracted were excluded from the analysis.

### 7. Repeat number of GT repetitive sequences in *HMOX1* promoter

The promoter region of the *HMOX1* gene was amplified by PCR using AmpliTaq Gold PCR Master Mix (Thermo Fisher Scientific) and specific primers with the following sequences: AGAGCCTGCAGCTTCTCAGA (forward) and ACAAAGTCTGGCCATAGGCA (reverse). The PCR reaction comprised preheating at 95°C for 10 min, 50 cycles of 95°C for 1 min, 60°C for 30 s, 70°C for 30 s, and a final extension at 70°C for 1.5 min. The PCR products were extracted using a NucleoSpin Gel and PCR clean-up kit (Takara Bio, Shiga, Japan). The number of GT repeats was determined from the sequences of PCR products analyzed by dye terminator methods using a DNA capillary sequencer (3730xl DNA analyzer, Thermo Fisher Scientific).

### 8. Oxidative stress level

As a parameter of oxidative stress levels, genomic DNA was pretreated by 8OHdG Assay Preparation Reagent Set (Wako Pure Chemical, Osaka, Japan), and 8OHdG levels were measured using the Highly Sensitive ELISA kit for 8OHdG (Japan Institute for the Control of Aging, Shizuoka, Japan) according to the manufacturers’ instructions. The absorbance at 450 nm was measured using a microplate reader (Beckman Coulter, Brea, CA, USA).

### 9. *HMOX1* mRNA expression level

The relative expression level of *HMOX1* was quantitatively analyzed by real-time reverse transcription polymerase chain reaction (RT-PCR), in which eukaryotic 18S rRNA was used as an internal control. Total RNA was reverse transcribed by a QuantiTect reverse transcription Kit (Qiagen). For real-time RT-PCR, the Mx3000P Real-time RT-PCR system (Agilent, Santa Clara, CA, USA), TaqMan Gene Expression Assay (HMOX1: Hs01110205_m1, Eukaryotic 18S rRNA Endogenous control: 4310413E, Thermo Fisher Scientific), and TaqMan Universal PCR Master Mix (Thermo Fisher Scientific) were used. The *HMOX1* mRNA expression level was quantified using the comparative Ct method. We excluded subjects whose Ct value of the internal standard was ≥36.

### 10. Statistical analysis

Descriptive data were expressed as the mean ± standard deviation (SD) for continuous variables and N (%) for categorical variables. All analyses were performed using SPSS Ver. 21.0 (IBM, Chicago, IL, USA). Values of *p* < 0.05 were considered statistically significant. To compare the basic attributes between obesity and non-obesity, and normal dermis and sparse dermis distinguished by ultrasonographic features, univariate analysis was conducted by unpaired Student’s *t*-tests or the Mann–Whitney *U*-test were used for continuous variables and chi-squared test or Fisher’s exact test for categorical variables. Fisher's exact test was conducted when there are 20% or more cells whose expected value of cells is less than 5 in contingency table. In subjects for which two types of alleles were measured, the relationship between numbers of GT repeats of longer alleles and *HMOX1* mRNA expression levels were analyzed by Spearman’s rank correlation coefficient. To determine the cut-off value for the number of GT repeats, we conducted a sensitivity analysis. The *HMOX1* mRNA expression levels were compared between two groups divided by the long (L) allele set from 25 to 30.

Differences in mRNA expression of *HMOX1* among three genotype groups were evaluated by the Kruskal–Wallis test followed by the Steel–Dwass test. After data were stratified for the presence or absence of obesity, the proportion of male subjects with L alleles was compared between normal distribution and sparse distribution of hyperechoic regions using ultrasonographic images.

### 11. Ethical considerations

This study was approved by the Human Genome and Genetic Analysis Ethics Committee of The University of Tokyo (G-3559-(1)) and the Kanazawa University (312). For this study, we used documents to explain the purpose and method of the survey to the subjects and obtained informed written consent.

## Results

### 1. Subjects

**Fig 2:**
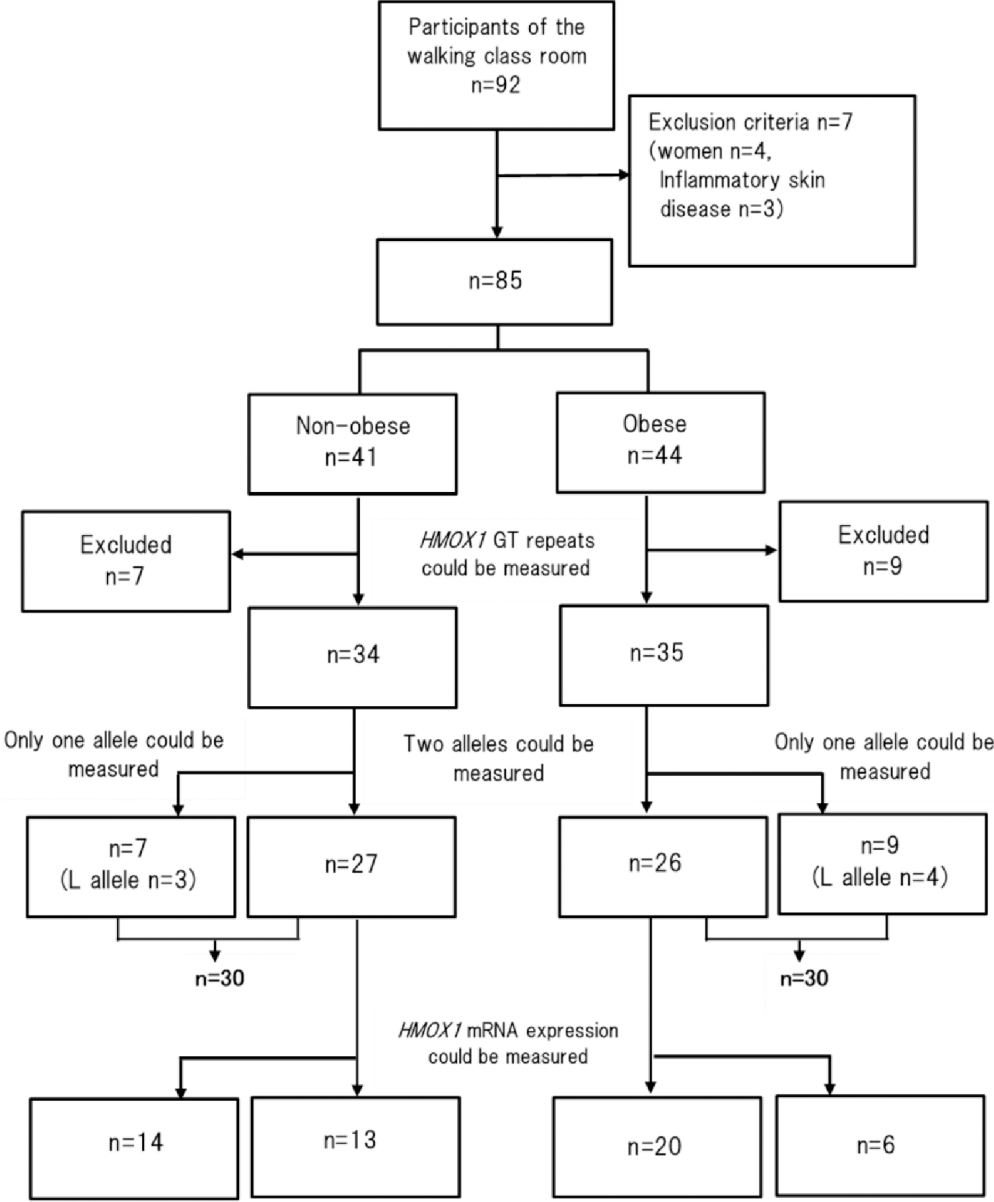
Flow of participants for each analysis.

The study flow of subjects through each analysis is shown in Fig 2. Overall, 92 subjects participated in the walking classroom. Female subjects and subjects with chronic inflammatory skin diseases (e.g., atopic dermatitis, psoriasis) were excluded. We excluded seven people who satisfied the exclusion criteria and received informed consent from 85 subjects. Overall, *HMOX1* polymorphism GT repeats were measured from the genomic DNA of 69 subjects, and both alleles were measured in 53 subjects. For the remaining 16 subjects, only one allele was decoded, of which seven subjects had an L allele. *HMOX1* mRNA expression levels were measured by real-time RT-PCR in 33 of 53 subjects only—it was not possible to measure either two types of allele and/or *HMOX1* mRNA expression levels in the other 20 subjects.

### 2. Characteristics of subjects

Characteristics of the 33 subjects are shown in Table 1. The age of the non-obese group tended to be lower than the obese group (*p* = 0.130). BMI (*p* < 0.01), body fat ratio (*p* < 0.01), and waist circumference (*p* < 0.01) were significantly higher in the obese group than in the control group. The proportion of subjects with sparse dermis in the obese group was significantly higher than in the non-obese group (*p <* 0.01). 8OHdG levels as an indicator of oxidative stress tended to be higher in the obese group compared with the non-obese group.

**Table 1:** Characteristics of 33 study subjects in whom two types of allele and *HMOX1* mRNA expression was measurable.

Items	Non-obese (*n* = 13)	Obese (*n* = 20)	*p*
Age (years)	36.38 (12.3)	40.40 (11.8)	0.130^b^
BMI (kg/m^2^)	21.69 (2.2)	27.62 (2.3)	<0.01^b^
Body fat ratio (%)	15.53 (4.7)	23.82 (4.2)	<0.01^b^
Waist circumference diameter (cm)	82.07 (8.8)	94.87 (6.6)	<0.01^a^
Smoking	4 (30.7)	4 (20.0)	0.681^c^
Diabetes^1^	0 (0.0)	1 (0.05)	0.606^c^
Hypertension^2^	0 (0.0)	3 (15.0)	0.209^c^
Hyperlipemia^3^	2 (15.4)	2 (10.0)	0.519^c^
Metabolic syndrome^4^	0 (0.0)	2 (10.0)	0.259^c^
Sparse dermis^5^	5 (38.5)	16 (80.0)	<0.01^d^
HMOX1 mRNA expression	0.069 (0.03)	0.062 (0.04)	0.285^b^
8 OHdG (mg/μL)	0.088 (0.05)	0.12 (0.05)	0.078^a^

^a^Student’s *t*-test

^b^Mann–Whitney *U*-test

^c^Fisher’s exact test

^d^Chi-square test.

Values are shown as the mean (SD) or n (%). 8OHdG, 8-hydroxy-2’-deoxyguanosine; BMI, body mass index; *HMOX1*, *heme oxygenase1*.

^1^Fasting blood glucose level ≥110 mg/dL and drug therapy.

^2^Systolic blood pressure ≥140 mmHg and/or diastolic blood pressure 90 mmHg and/or drug therapy.

^3^Serum triglyceride ≥150 mg/dL and/or HDL cholesterol < 40 mg/dL.

^4^Waist circumference diameter ≥85 cm and two or more of ^1–3^.

^5^Hyperechoic area is lacking part of, or the whole, dermis.

This survey was targeted at participants in a walking class for weight control or health promotion. Therefore, there the subjects of this study might have had a higher awareness of health compared with the general Japanese public. However, the mean BMI of the subjects in this study was 24.7 kg/m^2^, which is similar to the mean BMI of Japanese men (aged 30 to 40 years) [24]. Therefore, the subjects of this study are considered to represent the general Japanese population. There were no subjects who became non-obese from obese in the past 10 years.

### 3. Cut-off value of the number of GT repeats

**Fig 3:**
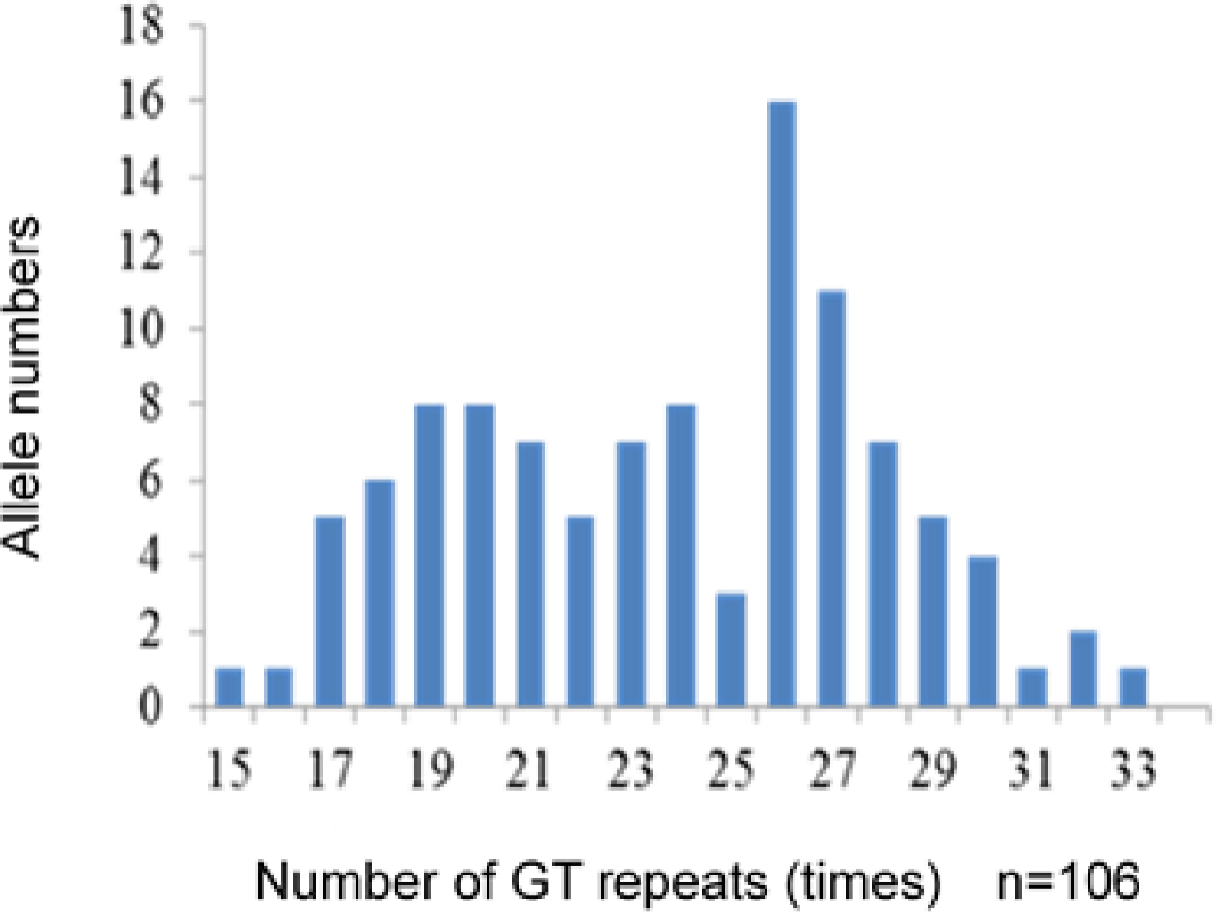
Distribution of alleles in subjects. The numbers of GT repeats in the *HMOX1* gene showed a distribution of 15–33 times. The distribution of the numbers of GT repeats was bimodal, with one peak located at 19–21 GT repeats and the other peak located at 26–27 GT repeats.

**Fig 4:**
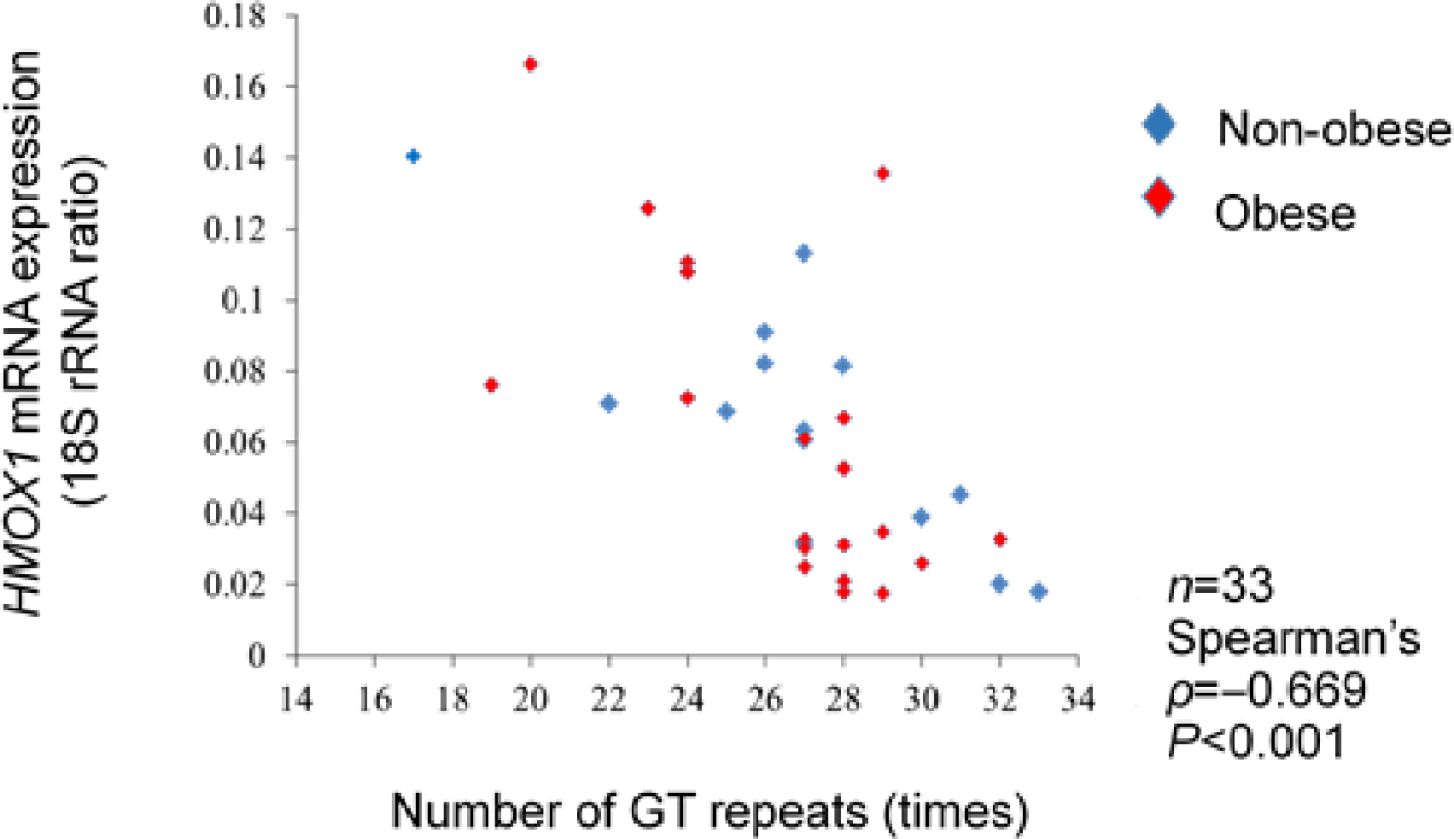
Correlation between the number of GT repeats and *HMOX1* mRNA expression levels. Among the two types of allele, the longer allele was included in the analysis. In 33 subjects, a significant negative correlation was observed between the GT repeat numbers and *HMOX1* mRNA expression levels (*ρ* = −0.669, *p* < 0.001).

The numbers of GT repeats in the *HMOX1* promoter ranged from 15 to 33 times and showed a bimodal distribution peak at 19–21 and 26–27 repeats (Fig 3). The GT repeat number of the longer allele was negatively correlated with *HMOX1* mRNA expression (*ρ* = −0.669, *p* = 0.01) (Fig 4).

In previous studies, the cut-off value to distinguish short and L alleles ranged from 25 to 30 repeats [25–28]. Therefore, we conducted sensitivity analysis at 25–30 times of GT repeats. *HMOX1* mRNA expression levels were significantly different between the L allele group and non-L allele group (0.101 ± 0.03 vs. 0.047 ± 0.03, *p* < 0.001) when the cut-off value of the number of GT repeats was set at 27 times. Because the z value was least at 27 times for GT repeats, we set the cut-off value at 27 times (Table 2).

**Table 2:** *HMOX1* expression level based on the cut-off value of the L allele.

Cut-off value	L allele	*n*	*HMOX1* mRNA expression level	*Z*	*p*
≥25	−	8	0.194 (0.04)	−3.36	0.001
	+	25	0.054 (0.03)		
≥26	−	9	0.104 (0.04)	−3.36	0.001
	+	24	0.050 (0.03)		
≥27	−	11	0.101 (0.03)	−3.82	<0.001
	+	22	0.047 (0.03)		
≥28	−	19	0.081 (0.04)	−2.73	0.006
	+	14	0.043 (0.03)		
≥29	−	25	0.070 (0.04)	−1.89	0.059
	+	8	0.042 (0.04)		
≥30	−	28	0.071 (0.04)	−1.91	0.056
	+	5	0.032 (0.01)		

Values are the mean (SD). Cut-off value is the times of GT repeat number. *P* value determined by the Mann–Whitney *U*-test.

### 4. Relationship between genotype and *HMOX1* mRNA expression

**Fig 5:**
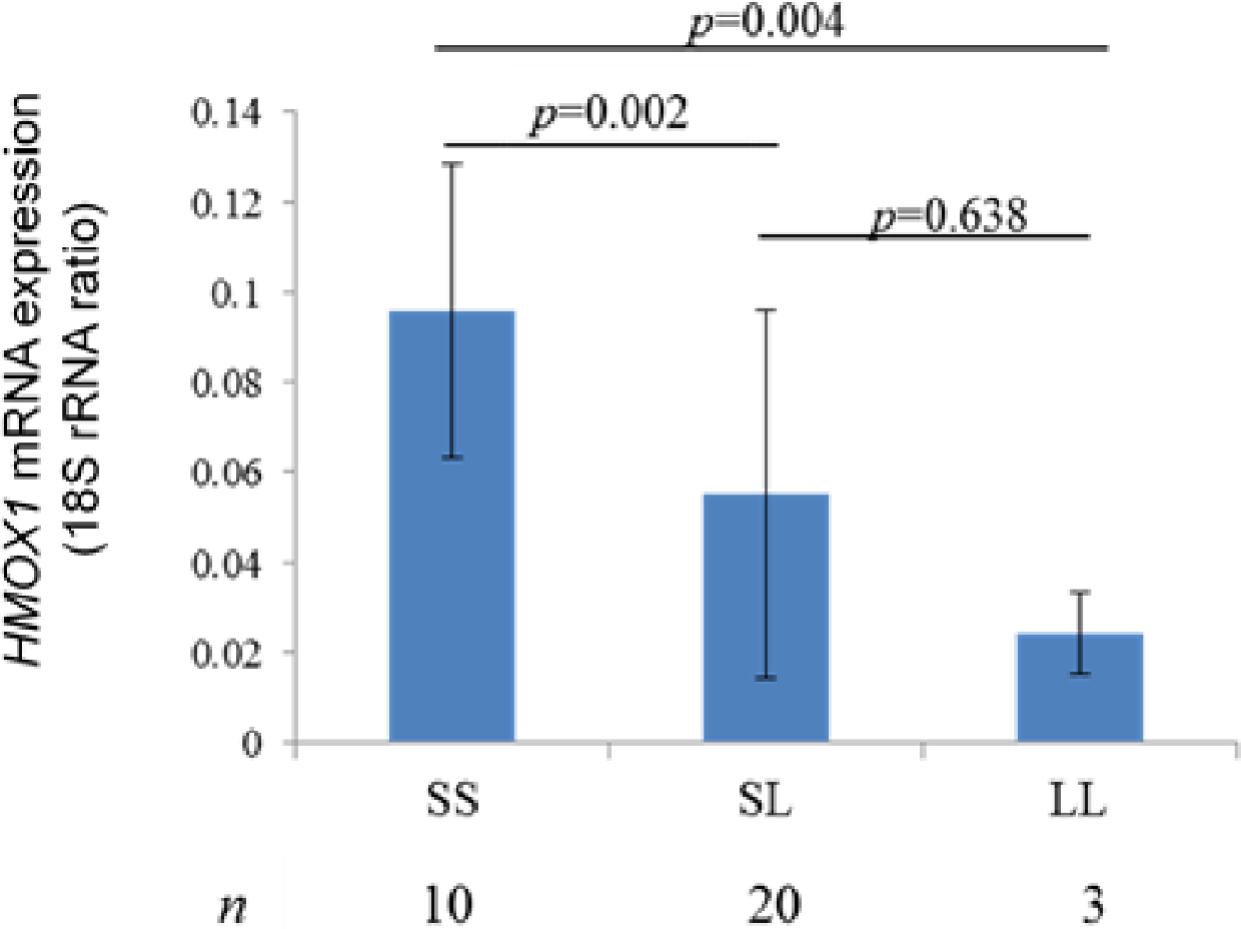
Relationship between genotype and *HMOX1* mRNA expression levels. Results of a comparison of each genotype and *HMOX1* mRNA expression levels were S/S = 0.01 ± 0.03, S/L = 0.049 ± 0.001, and L/L = 0.024 ± 0.009. S/S was significantly higher compared with S/L (*p* = 0.002) and L/L (*p* = 0.004).

Subjects for whom two alleles were measured were divided into S/S (10 subjects, 30.3%), S/L (20 subjects, 60.6%), and L/L (3 subjects, 9.1%) groups. *HMOX1* mRNA expression levels were significantly higher in the S/S (0.01 ± 0.03) group compared with S/L (0.049 ± 0.001, *p* = 0.002) and L/L (0.024 ± 0.009, *p* = 0.004) groups (Fig 5). These results showed that the presence of the L allele affected *HMOX1* mRNA expression levels. Therefore, this indicated that the dermal collagen structures of subjects were comparable between the L allele holders and non-holders.

### 5. Association of genotype with ultrasonographic features

In this analysis, 60 subjects (non-obese n = 30, obese n = 30) were included regardless of the measurement of *HMOX1* mRNA expression levels. The non-obese group consisted of 15 S/S subjects, 9 S/L subjects, 3 L/L subjects, and 3 subjects in which one of two alleles was identified as an L allele and the other was not identified (L/N). The obese group consisted of 11 S/S subjects, 14 S/L subjects, 1 L/L subject, and 4 L/N subjects. Characteristics of the 60 subjects are shown in Table 3. BMI, body fat ratio, waist circumference, and frequency of sparse dermis were significantly lower in the non-obese group than in the obese group. Age tended to decrease in the non-obese group compared with the obese group.

**Table 3:** Characteristics of 60 study subjects including those with only one measurable type of allele (L allele).

Items	Non-obese (*n* = 30)	Obese (*n* = 30)	*p*
Age (years)	36.56 (12.8)	41.93 (12.1)	0.073^b^
BMI (kg/m^2^)	22.17 (1.8)	27.29 (2.0)	<0.01^b^
Body fat ratio (%)	15.68 (4.4)	24.16 (3.8)	<0.01^b^
Waist circumference diameter (cm)	82.78 (6.7)	95.53 (6.4)	<0.01^a^
Smoking	10 (30.7)	7 (20.0)	0.567^d^
Diabetes	0 (0.0)	1 (0.05)	0.500^c^
Hypertension	1 (0.05)	3 (15.0)	0.612^c^
Hyperlipemia	2 (10.0)	2 (10.0)	0.694^c^
Metabolic syndrome	0 (0.0)	2 (10.0)	0.246^c^
Sparse dermis	16 (53.3)	24 (80.0)	0.050^d^
*HMOX1* mRNA expressions	0.066 (0.03)	0.064 (0.04)	0.526^b^
8 OHdG (mg/μL)	0.093 (0.05)	0.12 (0.05)	0.115^a^

^a^Student’s *t*-test

^b^Mann–Whitney *U*-test

^c^Fisher’s exact test

^d^Chi-square test.

Values are shown as the mean (SD) or n (%).

The relationship between the frequency of L allele holders and the classification of the ultrasonographic features were separately analyzed in the non-obese and the obese groups (Table 4). In the non-obese group, 6 of 14 normal dermis subjects (42.9%) and 9 of 16 sparse dermis subjects (56.3%) were L allele holders, but the difference of L allele holders was not significant for ultrasonographic features (*p* = 0.715). However, the frequency of L allele holders was significantly higher (18/24, 75.0%) in the sparse dermis compared with normal dermis subjects (1/6, 16.7%, *p* = 0.016) in the obese group.

**Table 4:** Relationship between ultrasound images and long allele holders.

		Distribution of hyperechoic signals, *n* (%)		
	*n*	Normal (n = 14)	Sparse dermis (n = 16)	*p*
S allele holder in the non-obese group	15	8 (57.1)	7 (43.7)	0.714^a^
L allele holder in the non-obese group	15	6 (42.9)	9 (56.3)	
		Distribution of hyperechoic signals, *n* (%)		
	*n*	Normal (n = 6)	Sparse dermis (n = 24)	*p*
S allele holder in the obese group	11	5 (83.3)	6 (25.0)	0.016^b^
L allele holder in the obese group	19	1 (16.7)	18 (75.0)	

*P* value determined by

^a^ Chi-square test or

^b^ Fisher’s exact test.

## Discussion

Recently, our research group revealed the oxidative stress-induced mechanical vulnerability of skin in obese subjects [4]. The current study provided additional findings that *HMOX1* gene polymorphisms cause individual differences in the expression capacity of an antioxidative enzyme, HMOX1, which affected the dermal collagen structure. It is expected that the examination of *HMOX1* polymorphisms might aid the identification of obese individuals with a risk of mechanical stress-related cutaneous wounds.

The cut-off values of the number of GT repeats ranged from 25 to 30 times in previous reports [25–28]. Similarly, we revealed a significantly higher *HMOX1* expression in body hair follicles when we set the cut-off value at 25–27 times of GT repeats. When the cut-off value was 27, *HMOX1* expression was approximately 2.15-fold higher in follicles from subjects without the L allele compared with those with the L allele, and the Z value was lowest with a cut-off value of 25–27 times. Our cut-off value agreed with the report of Hirai *et al*. [26], in which 2.01-time expression and 2.4-times activity of HMOX1 were observed in lymphoblasts without the L allele compared with cells with the L allele. However, this study evaluated the number of GT repeats in the *HMOX1* promoter and *HMOX1* mRNA expression level. Therefore, future studies will need to include an evaluation of HMOX 1 enzyme activity.

Previous studies reported that the prevalence of diabetes was associated with *HMOX1* polymorphisms [21]. It is also well known that the prevalence of diabetes is higher in obese people [29], and that hyperglycemia increases oxidative stress levels [30]. Therefore, it is essential to consider the effects of diabetes on the results in this study. Only one diabetic subject (5.4%) was enrolled in the obese group. Analysis excluding the diabetic subject showed similar results as when the subject was included (data not shown). Therefore, we considered the impact of diabetes was not significant in the present study.

The frequencies of L allele holders were not statistically different between the obese and the non-obese groups, suggesting no association of *HMOX1* promoter polymorphisms with obesity. However, further studies to clarify the relationship between *HMOX1* polymorphisms and obesity in different populations are warranted, because the mean BMI of subjects was relatively low (27.81 ± 2.39) in the obese group of this study.

The frequency of L allele holders was significantly higher in the sparse dermis group than in the normal dermis group among obese subjects. We previously revealed oxidative stress-dependent dermal collagen digestion in subcutaneous adipose tissues [4]. Nicolai *et al*. [31] reported that the cobalt protoporphyrin-induced increased expression of HMOX1 resulted in a reduction of oxidative stress and inflammatory responses in the subcutaneous adipose tissues of obese rats. Thus, our results suggested that the presence of the L allele might have resulted in the elevation of oxidative stress and dermal collagen digestion. Therefore, the presence of the L allele might be useful to identify persons at high risk of low collagen density. However, this study did not reveal the cause-relationship between the presence of the L allele and increased oxidative stress and dermal collagen digestion. Further research will be necessary.

There are several reports on the influence of HMOX1 expression on collagen metabolism. Yu *et al*. [32] reported that the increased activity of HMOX 1 by Smad 7 in cardiac fibroblasts suppressed collagen over synthesis because of the increased expression of Reactive oxygen species (ROS) / Matrix metalloproteinase (MMP) 9. Therefore, the increased expression of *HMOX1* by *HMOX1* polymorphisms in this study may suppress ROS in subcutaneous adipose tissue and increase the expression of MMP 2 and MMP 13, which degrade type 1 collagen, possibly causing low collagen density. Therefore, we believe that the evaluation of MMP gene expression in skin tissue is also important. However, the expression of MMPs is induced by various factors such as inflammatory cytokines other than ROS. In addition, because the *HMOX1* polymorphism was not affected by the current state and was always constant, it will be important to evaluate the *HMOX1* polymorphism.

To date, associations of the L allele with oxidative stress-related disorders are controversial. It was reported that the frequency of L allele holders was higher in chronic emphysema patients compared with non-patients among smokers [18], while the incidences of melanoma and psoriasis were not associated with the L allele of the *HMOX1* gene polymorphism [33, 34]. Similarly, in this study, the presence of the L allele was associated with alterations in dermal structures in obese subjects only. These results suggested that the association of *HMOX1* polymorphisms occurs during conditions of high oxidative stress.

The *HMOX1* gene polymorphism represents the antioxidant capacity of individuals. Therefore, this study clarified that the presence of the L allele of the *HMOX1* gene polymorphism in obese subjects was related to low collagen density. Thus, the examination of *HMOX1* gene polymorphisms might identify persons at high risk for low collagen density, which is a risk for pressure ulcers and skin tears caused by external forces in obese patients. Therefore, nurses can provide personalized skincare according to the individual risk level, resulting in the efficient prevention of skin impairment.

There were some limitations in this study. First, only male subjects were included. In women, the influence of estrogen was reported to have an impact on systemic oxidative stress levels [35]. Therefore, the analysis of female subjects considering the sexual cycle are required in the future. Second, the non-obese group tended to be younger than the obese group. It was suggested that the collagen density of skin decreases with age [36, 37]. Because differences in the proportion of subjects with low collagen density might be affected by age, further research including more subjects is required.

In conclusion, this study revealed that the presence of the L allele in obese individuals is associated with low collagen density. Therefore, the presence of *HMOX1* gene polymorphisms might identify persons at high risk for low collagen density, which is a risk for pressure ulcers and skin tears.
